# Effect of day-to-day variations in adrenal cortex hormone levels on abdominal symptoms

**DOI:** 10.1186/1751-0759-4-2

**Published:** 2010-03-18

**Authors:** Nagisa Sugaya, Shuhei Izawa, Namiko Ogawa, Kentaro Shirotsuki, Hitomi Kobayashi, Kosuke C Yamada, Hideki Tsumura, Shinobu Nomura, Hironori Shimada

**Affiliations:** 1 Faculty of Human Sciences, Waseda University, 2-579-15 Mikajima, Tokorozawa, Saitama 359-1192, Japan; 2 National Institute of Occupational Safety and Health, Health Administration and Psychosocial Factor Research Group, 6-21-1 Nagao, Tama-Ku, Kawasaki, Kanagawa 214-8585, Japan; 3 Graduate School of Human Sciences, Waseda University, 2-579-15 Mikajima, Tokorozawa, Saitama 359-1192, Japan; 4 Advanced Research Center for Human Sciences, Waseda University, 2-579-15 Mikajima, Tokorozawa, Saitama 359-1192, Japan

## Abstract

**Introduction:**

The hypothalamic-pituitary-adrenal axis is known to be related to abdominal symptoms, and the relationship between abdominal pain and cortisol secretory patterns has been previously investigated using a cross-sectional approach. Here, we investigated the effect of day-to-day variations in salivary cortisol and dehydroepiandrosterone-sulfate levels on abdominal symptoms in healthy individuals.

**Methods:**

Eleven college students (4 males and 7 females) participated in this study. The participants were asked to collect their saliva immediately after awakening and before bedtime for eight consecutive days. They also completed a questionnaire about abdominal symptoms before bedtime. The linear mixed model was applied to analyze the effects of the day-by-day variability or the 8-day average adrenal hormone level (at awakening, before bedtime, slope from awakening to bedtime) on abdominal symptoms.

**Results:**

The day-to-day variability of cortisol levels before bedtime was negatively related with loose stool, while the day-to-day variability of the cortisol slope was positively correlated with loose stool. A low 8-day average dehydroepiandrosterone-sulfate level at awakening was positively related with frequent bowel movements, loose stool, and long bouts of severe abdominal pain. Likewise, a low 8-day average dehydroepiandrosterone-sulfate slope was positively related with long bouts of abdominal pain.

**Conclusions:**

Low cortisol levels before bedtime and a steeper diurnal cortisol slope during the day may be related to bouts of diarrhea during the day.

## Introduction

The effect of changes in the hypothalamic-pituitary-adrenal (HPA) axis on abdominal symptoms has been previously reported. On the basis of the results of previous studies, Mayer et al. (2002) suggested that complex interrelationships exist between gut-associated immune tissue, the central nervous system, and the enteric nervous system [1]. Psychosocial stressors activate stress circuits within the emotional motor system, and the resulting peripheral output is manifested in the form of cortisol, corticotrophin-releasing factor, and autonomic (norepinephrine and epinephrine) responses, which induces the mucosal immune system to activate a Th2 response [2,3]. An increase in mast cells brought about by alterations in the Th1/Th2 balance may stimulate bowel movements by altering the access of luminal organisms and antigens to the gut immune system [4,5].

Previous studies have suggested a relationship between abdominal pain and the cortisol response levels in the morning and evening. Patients with functional gastrointestinal disorder have been shown to have lower cortisol levels 60 min after awakening as compared with healthy controls although there was no significant difference in the cortisol response after awakening between these groups. Furthermore, total secretion (area under the curve) of cortisol during the first hour after awakening was also found to be lower in patients with functional gastrointestinal disorder than healthy controls [6]. Compared with controls, patients with irritable bowel syndrome (IBS) showed significantly higher cortisol levels in the morning and lower levels in the evening while maintaining the physiological circadian fluctuation (i.e., morning cortisol levels higher than in the evening) [7]. Moreover, a previous study suggested that in children with recurrent abdominal pain of psychosomatic origin, levels of cortisol in the morning and total secretion were significantly higher than those in healthy children [8]. Another study reported that children with recurrent abdominal pain showed higher cortisol levels in the morning than healthy children [9]. Although these studies do not present consistent findings, it is likely that there exists a relationship between cortisol secretion and abdominal symptoms. Indeed, the previous studies clearly show that there is a difference in cortisol secretion between groups with and without functional gastrointestinal symptoms.

It has been reported that variations in the HPA axis marker have an impact on abdominal symptoms, and the relationship between abdominal pain and cortisol secretory patterns has been investigated using the cross-sectional approach. However, the following problems must be addressed. First, in addition to investigating the effect of cortisol secretory patterns on abdominal symptoms, we must examine day-to-day intraindividual variations in cortisol secretion in relation to the severity of abdominal symptoms. Second, it is important to clearly elucidate the relationship between abdominal symptoms and dehydroepiandrosterone (DHEA). DHEA is the most important androgen secreted by the adrenal cortex and is a precursor for sex steroids; the main secretagogue for DHEA is thought to be adrenocorticotrophic hormone. DHEA is thought to affect immunological function in a manner opposite to that of cortisol [10]. DHEA is converted into DHEA-sulfate (DHEA-S) and accumulated within a relatively short time after secretion. Therefore, DHEA-S is the major circulating form, and is converted to DHEA by a sulfotransferase in the tissue. In this study, we collected saliva after relatively long intervals to determine DHEA-S, which has an apulsatile secretion pattern and which was more useful for our study purposes than DHEA, which has a pulsatile secretion pattern. Thus, analysis of both cortisol and DHEA-S may be useful with regard to studying the effects of adrenal hormones on abdominal symptoms.

We aimed to examine the relationship between physiological factors and abdominal symptoms by assessing day-to-day intraindividual variations in cortisol and DHEA-S secretion patterns. For this, we investigated the effect of daily changes in adrenal hormone (salivary cortisol and DHEA-S) levels on abdominal symptoms by monitoring the subjects for eight days.

## Methods

### Participants

The participants were 11 college students (4 males and 7 females, mean age = 26.9 ± 2.8 yrs). Mean body mass index (BMI) of the participants was 20.9 kg/m^2^. No one used any habitual medications or dietary supplements that affect HPA activity or had any history of gastrointestinal surgery for the past five years. Written informed consent was obtained, and the study was approved by the university's ethics committee.

### Measures (diaristic questionnaire)

#### 1) Information related to abdominal symptoms

The following items were assessed: "Bowel movement," "Stool properties," "Duration of abdominal pain (minutes)," and "Degree of abdominal pain."

"Bowel movement" was assessed by asking the following question: "How many bowel movements did you have today?;" "Stool property" was based on a rating of 1 to 8 (1: no bowel movement; 2: lumpy stool; 3: hard stool; 4: like a banana with cracks on its surface; 5: like a banana, smooth and soft; 6: soft blobs with clear-cut edges; 7: fluffy pieces with ragged edges, mushy; 8: watery, no solid pieces); "Duration of abdominal pain" was assessed by asking the following question: "How long did you experience continuous abdominal pain today?;" and "Degree of abdominal pain" was rated from 0 (not at all) to 10 (very severe).

#### 2) Information about sleep duration

Sleep duration was calculated from the time subjects went to sleep to the time they awakened. Previous research has noted a correlation between cortisol secretion levels and sleep duration [11].

#### 3) Perceived Stress Scale

The Perceived Stress Scale 4 (PSS4) [12,13] was designed to measure the degree to which situations in one's life are appraised as stressful. The PSS4 includes four items, each of which are rated from 0 to 4. In this study, the PSS4 was used in order to consider the effect of psychological stress on adrenal hormone secretion.

### Determination of adrenal hormone levels

Participants were asked to collect their saliva samples at awakening and immediately before bedtime for eight consecutive days. The samples were used to determine cortisol and DHEA-S levels. Saliva samples used to determine cortisol levels were collected using Salivette swabs (Sarstedt Ltd., Germany.). For samples for determining DHEA-S levels patients were asked to expectorate saliva through a short plastic straw into a collection vial. Saliva samples were stored at room temperature (10-20°C) and centrifuged at 3000 rpm for 5 min within three days after sampling. The concentrations of cortisol and DHEA-S in the saliva were determined by an enzyme immunoassay using an EIA Kit (Cortisol: Salimetrics LLC, USA; DHEA-S: Diagnostic Systems Laboratories, Inc., USA).

### Procedure

Participants were asked to collect their saliva on awakening and immediately before bedtime. They were also asked to complete the diaristic questionnaire before bedtime for eight consecutive days. The participants were asked to not drink alcohol during the experimental period. Further, we avoided selecting female subjects who were menstruating during the period so that menstrual pain would not be confused with bowel pains.

### Statistical Analysis

For the analysis of adrenal hormone data, cortisol and DHEA-S concentration values were square root transformed. The relationship between adrenal hormone, abdominal symptoms, stress, and sleep duration was analyzed using Pearson's product-moment correlation coefficient. Student's *t *test for repeated measures was applied to analyze the cortisol and DHEA-S slopes between awakening and bedtime. The linear mixed model was applied to analyze the effect of adrenal hormones on abdominal symptoms. The abdominal symptoms were dependent variables and the 8-day average adrenal hormone level, the daily intraindividual variability of adrenal hormones (at awakening, before bedtime, slope from awakening to bedtime [the value at awakening minus the value before bedtime]), the PSS4 score, and the sleep duration were covariates. Data analysis was performed using the SPSS 15.0 software (SPSS Japan Inc.).

## Results

### Basal Attribution

The mean sleep duration was 5.67 ± 1.56 hours. The mean PSS4 score was 5.97 ± 2.80. As shown in Table 1, adrenal hormone levels at awakening were significantly higher than those before bedtime (cortisol: *t *= 11.91, *p *< 0.001; DHEA-S: *t *= 9.21, *p *< 0.001).

**Table 1:** Comparison of adrenal hormone levels at awakening and before bedtime

	At awakening	Before bedtime		
				
	Mean Score	Mean Score	*t*	
Cortisol	2.77 ± 0.79	1.29 ± 0.74	11.91	***
DHEA-S	3.78 ± 1.48	2.23 ± 1.37	9.21	***

*** *p *< 0.001

Cortisol and DHEA-S concentration values were square root transformed.

### Correlations among adrenal hormones, stress, and sleep duration

As shown in Table 2, sleep duration was positively correlated with the PSS4 score (*r *= 0.34, *p *< 0.001) and bowel movement (*r *= -0.26, *p *< 0.01). Cortisol levels on awakening were positively correlated with sleep duration (*r *= 0.24, *p *< 0.05) and stool property (*r *= 0.21, *p *< 0.05). Cortisol levels before bedtime were negatively correlated with sleep duration (*r *= -0.22, *p *< 0.05) and stool property (*r *= -0.36, *p *< 0.001). The cortisol slope from awakening to bedtime was positively correlated with sleep duration (*r *= 0.32, *p *< 0.01) and stool property (*r *= 0.31, *p *< 0.01).

**Table 2:** Correlation among adrenal hormone levels, stress, and sleep duration

			At awakening		Before bedtime		Slope from awakening to before bedtime	
					
	PSS	Sleep duratoin	Cortisol	DHEA-S	Cortisol	DHEA-S	Cortisol	DHEA-S
PSS	-	-	0.01	-0.31 **	-0.05	-0.37 ***	0.04	0.05
Sleep duration	0.34 ***	-	0.24 *	-0.33 **	-0.22 **	-0.35 ***	0.32 **	0.00
Bowel movement	0.10	0.26 **	0.08	-0.40 ***	-0.11	-0.30 **	0.12	-0.11
Stool property	-0.12	0.11	0.21	-0.39 ***	-0.36 ***	-0.34 **	0.31 **	-0.14
Duration of abdominal pain	-0.02	0.09	0.11	-0.14	-0.09	-0.09	0.13	-0.06
Degree of abdominal pain	-0.11	0.07	0.00	-0.26 *	-0.12	-0.23 *	0.05	-0.20

*** *p *< 0.001 ** *p *< 0.01 * *p *< 0.05

DHEA-S levels at awakening and before bedtime were negatively correlated with the PSS4 score (at awakening: *r *= -0.31, *p *< 0.01; before bedtime: *r *= -0.37, *p *< 0.001), sleep duration (at awakening: *r *= -0.33, *p *< 0.01; before bedtime: *r *= -0.35, *p *< 0.001), bowel movement (at awakening: *r *= -0.40, *p *< 0.001; before bedtime: *r *= -0.30, *p *< 0.01), stool property (at awakening: *r *= -0.39, *p *< 0.001; before bedtime: *r *= -0.34, *p *< 0.01), and the degree of abdominal pain (at awakening: *r *= -0.26, *p *< 0.05; before bedtime: *r *= -0.23, *p *< 0.05).

### Effect of adrenal hormone levels at awakening on abdominal symptoms

The results of the linear mixed model with abdominal symptoms as dependent variables and the day-to-day intraindividual variability, the 8-day average adrenal hormone levels at awakening, the PSS4 score, and sleep duration as covariates are shown in Table 3. The 8-day average DHEA-S level at awakening was negatively related to bowel movement (*t *= -3.31, *p *< 0.05), stool property *(t *= -2.64, *p *< 0.05), the duration of abdominal pain (*t *= -2.59, *p *< 0.05), and the degree of abdominal pain (*t *= -2.37, *p *< 0.05).

**Table 3:** Effect of adrenal hormone levels at awakening on abdominal symptoms

*Bowel movement*		Estimate	SE	*t*	*p*	*Duration of abdominal pain*		Estimate	SE	*t*	*p*
**Average**	Cortisol	0.42	0.30	1.42	0.20	**Average**	Cortisol	11.52	14.03	0.82	0.41
	DHEA-S	-0.39	0.12	-3.31	0.02 *		DHEA-S	-14.22	5.49	-2.59	0.01 *
	Sleep duration	-0.25	0.20	-1.22	0.27		Sleep duration	-15.71	9.60	-1.64	0.11
	PSS	0.08	0.07	1.14	0.30		PSS	1.60	3.30	0.48	0.63

**Variability**	Cortisol	0.04	0.12	0.35	0.73	**Variability**	Cortisol	6.70	6.23	1.07	0.29
	DHEA-S	-0.06	0.09	-0.69	0.49		DHEA-S	0.06	4.74	0.01	0.99
	Sleep duration	-0.05	0.07	-0.67	0.50		Sleep duration	-2.10	3.49	-0.60	0.55
	PSS	-0.04	0.04	-0.84	0.40		PSS	-1.55	2.26	-0.68	0.50

***Stool property***		Estimate	SE	*t*	*p*	***Degree of abdominal pain***		Estimate	SE	*t*	*p*

**Average**	Cortisol	0.15	0.93	0.16	0.88	**Average**	Cortisol	0.17	0.44	0.40	0.69
	DHEA-S	-0.96	0.36	-2.64	0.04 *		DHEA-S	-0.40	0.17	-2.37	0.02 *
	Sleep duration	-0.35	0.64	-0.55	0.60		Sleep duration	-0.02	0.30	-0.06	0.95
	PSS	-0.13	0.22	-0.61	0.57		PSS	-0.04	0.10	-0.38	0.71

**Variability**	Cortisol	0.30	0.27	1.10	0.28	**Variability**	Cortisol	-0.04	0.19	-0.22	0.83
	DHEA-S	-0.35	0.21	-1.68	0.10		DHEA-S	-0.07	0.15	-0.48	0.63
	Sleep duration	-0.11	0.15	-0.74	0.46		Sleep duration	-0.06	0.11	-0.57	0.57
	PSS	-0.09	0.10	-0.90	0.37		PSS	-0.08	0.07	-1.09	0.28

* *p *< 0.05

### The effect of adrenal hormone levels before bedtime on abdominal symptoms

The results of the linear mixed model with abdominal symptoms as dependent variables and the day-to-day intraindividual variability, the 8-day average adrenal hormone levels before bedtime, the PSS4 score, and sleep duration as covariates are shown in Table 4. The day-to-day variability of cortisol level before bedtime was negatively related to stool property (*t *= -2.13, *p *< 0.05), while the 8-day average DHEAS level was negatively related to bowel movement (*t *= -2.78, *p *< 0.05) and stool property (*t *= -2.42, *p *< 0.05).

**Table 4:** Effect of adrenal hormone levels before bedtime on abdominal symptoms

*Bowel movement*		Estimate	SE	*t*	*p*	*Duration of abdominal pain*		Estimate	SE	*t*	*p*
**Average**	Cortisol	0.49	0.31	1.57	0.16	**Average**	Cortisol	4.70	15.27	0.31	0.77
	DHEA-S	-0.46	0.17	-2.78	0.03 *		DHEA-S	-8.24	8.07	-1.02	0.34
	Sleep duration	-0.05	0.19	-0.25	0.81		Sleep duration	-5.94	9.33	-0.64	0.55
	PSS	0.02	0.07	0.25	0.81		PSS	0.22	3.54	0.06	0.95

**Variability**	Cortisol	-0.05	0.14	-0.37	0.72	**Variability**	Cortisol	-4.68	7.45	-0.63	0.53
	DHEA-S	0.01	0.10	0.09	0.93		DHEA-S	-1.16	5.22	-0.22	0.83
	Sleep duration	-0.02	0.07	-0.36	0.72		Sleep duration	-2.70	3.58	-0.75	0.45
	PSS	-0.04	0.04	-0.81	0.42		PSS	-1.36	2.32	-0.58	0.56

***Stool property***		Estimate	SE	*t*	*p*	***Degree of abdominal pain***		Estimate	SE	*t*	*p*

**Average**	Cortisol	0.44	0.88	0.50	0.64	**Average**	Cortisol	0.17	0.43	0.39	0.71
	DHEA-S	-1.12	0.46	-2.41	0.049 *		DHEA-S	-0.31	0.23	-1.34	0.22
	Sleep duration	-0.35	0.55	-0.64	0.54		Sleep duration	0.16	0.26	0.60	0.57
	PSS	-0.10	0.21	-0.48	0.65		PSS	-0.06	0.10	-0.55	0.60

**Variability**	Cortisol	-0.65	0.32	-2.07	0.04 *	**Variability**	Cortisol	-0.10	0.23	-0.44	0.66
	DHEA-S	0.05	0.22	0.23	0.82		DHEA-S	-0.08	0.16	-0.49	0.63
	Sleep duration	-0.02	0.15	-0.14	0.89		Sleep duration	-0.04	0.11	-0.36	0.72
	PSS	-0.09	0.10	-0.92	0.36		PSS	-0.09	0.07	-1.22	0.23

* *p *< 0.05

### Effect of adrenal hormone slopes from awakening to bedtime on abdominal symptoms

The results of the linear mixed model with abdominal symptoms as dependent variables and the day-to-day intraindividual variability, the 8-day average adrenal hormone slopes, the PSS4 score, and sleep duration as covariates are shown in Table 5. The day-to-day variability of the cortisol slope was positively related to stool property (*t *= 2.07, *p *< 0.05), while the 8-day average DHEA-S slope was negatively related to the duration of abdominal pain (*t *= -2.07, *p *< 0.05).

**Table 5:** Effect of adrenal hormone slopes from awakening to bedtime on abdominal symptoms

*Bowel movement*		Estimate	SE	*t*	*p*	*Duration of abdominal pain*		Estimate	SE	*t*	*p*
**Average**	Cortisol	0.28	0.47	0.59	0.58	**Average**	Cortisol	22.52	13.21	1.70	0.09
	DHEA-S	-0.18	0.27	-0.69	0.52		DHEA-S	-15.65	7.56	-2.07	0.04 *
	Sleep duration	0.06	0.35	0.18	0.87		Sleep duration	-12.67	9.94	-1.27	0.21
	PSS	0.08	0.13	0.62	0.56		PSS	3.56	3.75	0.95	0.35

**Variability**	Cortisol	0.05	0.09	0.55	0.58	**Variability**	Cortisol	4.48	4.43	1.01	0.32
	DHEA-S	-0.03	0.06	-0.53	0.60		DHEA-S	0.27	3.21	0.08	0.93
	Sleep duration	-0.03	0.07	-0.40	0.69		Sleep duration	-2.96	3.61	-0.82	0.41
	PSS	-0.04	0.05	-0.78	0.44		PSS	-1.38	2.34	-0.59	0.56

***Stool property***		Estimate	SE	*t*	*p*	***Degree of abdominal pain***		Estimate	SE	*t*	*p*

**Average**	Cortisol	0.97	1.28	0.76	0.48	**Average**	Cortisol	0.52	0.41	1.28	0.21
	DHEA-S	-0.48	0.73	-0.66	0.53		DHEA-S	-0.37	0.24	-1.58	0.12
	Sleep duration	-0.03	0.97	-0.03	0.98		Sleep duration	0.09	0.31	0.30	0.77
	PSS	0.05	0.36	0.13	0.90		PSS	0.02	0.12	0.13	0.90

**Variability**	Cortisol	0.40	0.19	2.13	0.04 *	**Variability**	Cortisol	0.00	0.14	0.03	0.97
	DHEA-S	-0.18	0.14	-1.36	0.18		DHEA-S	-0.01	0.10	-0.05	0.96
	Sleep duration	-0.02	0.16	-0.12	0.90		Sleep duration	-0.07	0.11	-0.67	0.51
	PSS	-0.08	0.10	-0.85	0.40		PSS	-0.08	0.07	-1.03	0.31

* *p *< 0.05

## Discussion

This is the first study to investigate the effect of the day-to-day variability of adrenal hormone levels on abdominal symptoms in healthy individuals. There is a possibility that a steeper diurnal cortisol slope during the day and lower cortisol levels in the evening is related to bouts of diarrhea. The results of this study are similar to those of two previous studies [7,14]. Previous research suggests that compared with control patients, IBS patients show significantly higher levels of cortisol in the morning and lower levels in the evening [7]. Therefore, the cortisol secretion patterns influencing vulnerability to abdominal symptoms may be similar in healthy individuals and individuals with IBS. Moreover, Sugaya et al. [14] reported that individuals with IBS had lower cortisol responses under acute stress. The result of this study is similar to that of Sugaya et al. [14] with respect to the relationship between the lower cortisol response and abdominal symptoms. The results of the present study are similar to those of Patachioli et al. [7] and Sugaya et al. [14]; however, the analysis of the day-to-day variability of cortisol provided a more definitive conclusion. The previous studies only indicated the characteristic of cortisol at a specific point in time. From these results, we indicate the continuity between healthy individuals and individuals with IBS with respect to the relationship between cortisol and abdominal symptoms.

We found no significant effects of day-to-day variability of DHEA-S on bowel symptoms. However, the 8-day average DHEA-S level at awakening was negatively related to all variables of abdominal symptoms. These results suggest that chronically reduced levels of DHEA-S at awakening or baseline levels are related to the degree and duration of abdominal pain, diarrhea, and infrequent bowel movements. In addition, the 8-day average of the DHEA-S slope was related to the duration of abdominal pain. Therefore, a flat circadian rhythm of DHEA-S can increase the duration or frequency of abdominal pain. Moreover, the 8-day average DHEA-S level before bedtime was negatively related to the frequency of bowel movements and stool property. In fact, we assert that low DHEA-S levels may have an affect on the frequency of bowel movements and stool property to a higher degree than the slope between the morning and the evening.

Previous research has suggested the possibility that higher cortisol and lower DHEA (or DHEA-S) levels may result in altered immune function, which in turn may cause abdominal pain [2,5,10]. However, our findings do not suggest that higher cortisol levels affect abdominal symptoms. Sugaya et al. [14] indicated that the secretion of a secretagogue, which induces the production of an adrenal hormone (e.g., adrenocorticotrophic hormone and corticotrophine-releasing hormone), may be normal in individuals with IBS because they showed a lower cortisol response than healthy controls and their DHEA profiles were similar to those of healthy controls. Therefore, considering the results of the present study and those of Sugaya et al. [14], there is a possibility that lower cortisol levels may aggravate abdominal symptoms due to a dysfunction of negative feedback regulation to corticoprophin-releasing hormone secretion. We can speculate that chronically low DHEA-S levels may cause the deterioration of abdominal pain and diarrhea with respect to a decreased effect of DHEA-S on immunological function that is opposite to cortisol.

This study provides some new insights and provides valuable information for future research; nevertheless, there were three limitations to the present work. First, we were unable to consider the relation between the participants dietary habits and their abdominal symptoms. Although inclusion of items about dietary habits may yield more accurate results, we feel that future studies should employ a less demanding saliva sampling process over the 8-day period. Second, the diurnal cortisol slope was calculated by only determining cortisol levels immediately after awakening and before bedtime; samples were not obtained for any other times. Hence, our results may not be useful for determining the cause of abdominal pain taking place early in the day. In the future, the relationship between the circadian rhythm of adrenal hormones and abdominal symptoms must be determined by taking multiple saliva samples throughout the day. Therefore, future studies should aim to maintain a good balance between consideration for the participants and collecting multiple samples. Third, the number of participants in this study was rather small.

## Conclusions

This is the first study to investigate the effects of day-to-day variations in salivary cortisol and DHEA-S secretion on abdominal symptoms. The results of our study suggest that variations in adrenal hormone levels affect the duration and severity of abdominal symptoms. Day-to-day variations in cortisol levels before bedtime and a steeper cortisol slope may be positively related to a tendency toward bouts of diarrhea. On the other hand, a persistent lower baseline and a flatter slope with regard to DHEA-S may also be negatively related to abdominal symptoms. The results of our study suggested the possibility that in the future the accurate clinical condition of abdominal symptoms may be able to be revealed by a device that can take multiple saliva samples throughout the day and by better controlling various daily habits.

## Competing interests

The authors declare that they have no competing interests.

## Authors' contributions

NS conceptualized and designed the study, collected the data, conducted the saliva analysis, performed the statistical analysis, and drafted the manuscript. SI, NO, KS, HK, KY, and HT conceptualized and designed the study, collected the data, and conducted the saliva analysis. SN and HS conceptualized and designed the study. All authors read and approved the final manuscript.
